# C/EBP-Family Redundancy Determines Patient Survival and Lymph Node Involvement in PDAC

**DOI:** 10.3390/ijms24021537

**Published:** 2023-01-12

**Authors:** Leonie Hartl, Joris J. T. H. Roelofs, Frederike Dijk, Maarten F. Bijlsma, JanWillem Duitman, C. Arnold Spek

**Affiliations:** 1 Laboratory for Experimental Oncology and Radiobiology, Center for Experimental and Molecular Medicine, Amsterdam UMC Location University of Amsterdam, 1105 AZ Amsterdam, The Netherlands; 2 Cancer Center Amsterdam, Cancer Biology and Immunology, 1081 HV Amsterdam, The Netherlands; 3 Department of Pathology, Amsterdam UMC Location University of Amsterdam, 1105 AZ Amsterdam, The Netherlands; 4 Department of Pulmonary Medicine, Amsterdam UMC Location University of Amsterdam, 1105 AZ Amsterdam, The Netherlands; 5 Department of Experimental Immunology, Amsterdam UMC Location University of Amsterdam, 1105 AZ Amsterdam, The Netherlands; 6 Amsterdam Infection & Immunity, Inflammatory Diseases, 1105 AZ Amsterdam, The Netherlands

**Keywords:** C/EBP, *CEBPB*, *CEBPD*, *CEBPG*, redundancy, PDAC, lymph node involvement

## Abstract

Pancreatic ductal adenocarcinoma (PDAC) is a dismal disease with a poor clinical prognosis and unsatisfactory treatment options. We previously found that the transcription factor CCAAT/Enhancer-Binding Protein Delta (C/EBPδ) is lowly expressed in PDAC compared to healthy pancreas duct cells, and that patient survival and lymph node involvement in PDAC is correlated with the expression of C/EBPδ in primary tumor cells. C/EBPδ shares a homologous DNA-binding sequence with other C/EBP-proteins, leading to the presumption that other C/EBP-family members might act redundantly and compensate for the loss of C/EBPδ. This implies that patient stratification could be improved when expression levels of multiple C/EBP-family members are considered simultaneously. In this study, we assessed whether the quantification of C/EBPβ or C/EBPγ in addition to that of C/EBPδ might improve the prediction of patient survival and lymph node involvement using a cohort of 68 resectable PDAC patients. Using Kaplan–Meier analyses of patient groups with different C/EBP-expression levels, we found that both C/EBPβ and C/EBPγ can partially compensate for low C/EBPδ and improve patient survival. Further, we uncovered C/EBPβ as a novel predictor of a decreased likelihood of lymph node involvement in PDAC, and found that C/EBPβ and C/EBPδ can compensate for the lack of each other in order to reduce the risk of lymph node involvement. C/EBPγ, on the other hand, appears to promote lymph node involvement in the absence of C/EBPδ. Altogether, our results show that the redundancy of C/EBP-family members might have a profound influence on clinical prognoses and that the expression of both C/EPBβ and C/EBPγ should be taken into account when dichotomizing patients according to C/EBPδ expression.

## 1. Introduction

Pancreatic ductal adenocarcinoma (PDAC) is a dismal disease with an extremely low 5-year survival rate of only 11% and a continuously increasing incidence in developed countries [1]. Since primary ductal adenocarcinomas of the pancreas do not cause clinical symptoms in the early phase, the disease is typically detected at an advanced stage. At the time of diagnosis, cells have often metastasized to distant organs, precluding curative resection. To date, no curative treatment for metastatic PDAC has been found, making it a particularly lethal disease and the predicted second-leading cause of cancer-related death by 2030 [2].

CCAAT/Enhancer-Binding Protein Delta (C/EBPδ) is a member of the family of CCAAT/enhancer-binding protein transcription factors that plays key roles in differentiation, cell cycle regulation, proliferation, and apoptosis in healthy tissue development, during inflammation and in cancer [3,4,5]. In cancer, C/EBPδ appears to play a highly context-dependent role [6]. On one hand, C/EBPδ has been shown to function as a tumor suppressor through the induction of apoptosis in hepatocellular carcinoma cell lines [7,8], growth inhibition in acute myeloid leukemia [9], and suppression of SNAI2 expression in breast cancer [10]. On the other hand, C/EBPδ promotes metastasis formation in urothelial carcinoma [11], tumor growth of hepatocellular carcinoma xenografts [12], and hypoxia-adaptation in glioblastoma [13] and breast cancer [14]. In PDAC, C/EBPδ acts as a suppressor of tumorigenesis by limiting cell proliferation, clonogenicity and motility [15,16]. Accordingly, patients with tumor cells expressing high levels of C/EBPδ show a significantly longer survival and a lower likelihood of lymph node involvement, i.e., presence of tumor cells in sentinel and surrounding lymph nodes, than patients whose primary tumor cells express low levels of C/EBPδ [15].

C/EBP-family members share a highly homologous C-terminal domain that comprises a basic DNA-binding region and four-to-five leucine residues—the basic leucine zipper (bZIP)—that allow dimerization of the C/EBP- and bZIP-families with other transcription factors [17]. Due to the conserved nature of the DNA-binding domain, all C/EBP-family members bind to identical DNA recognition sites in the promoter regions of C/EBP-target genes [18], which leads to a transcriptional redundancy among some of the members. This has, for instance, been shown in the context of IL-1 and IL-6 induction but also during adipocyte differentiation and in acute myeloid leukemia, where the loss of a C/EBP family member is—at least in part—compensated for by its siblings [19,20,21]. Next to this, C/EBP-members are often collectively regulated as has been shown during the acute phase response and in acute myeloid leukemia [22,23], further implying a synergistic behavior and potential redundancy of C/EBP-family members. This redundancy and the consequent compensatory mechanisms would suggest that stratification of patients by expression levels of a single family member might be suboptimal and could be improved when expression levels of other C/EBP-family members are also considered. To test this hypothesis, we followed up on our previous data showing that C/EBPδ expression is associated with survival and lymph node involvement in PDAC patients [15]. Specifically, we investigated whether, along with C/EBPδ, other C/EBP-family members might act as predictors of patient survival and lymph node involvement and whether the quantification of other family members in addition to that of C/EBPδ might improve the prediction of survival and lymph node involvement in PDAC.

## 2. Results

### 2.1. CEBP-Family Member Expression in PDAC

**Figure 1:**
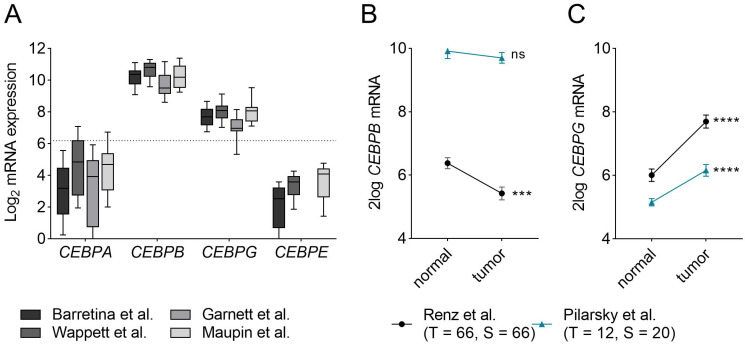
mRNA expression of *CEBP*-members in PDAC. (**A**) mRNA expression of *CEBPA*, *CEBPB*, *CEBPG* and *CEBPE* in different PDAC cell line panels published by Barrettina et al. (*n*= 43) [26], Garnett et al. (*n* = 17) [27], Wappett et al. (*n* = 41) [28] and Maupin et al. (*n* = 22) [29]. While *CEBPA* and *CEBPE* are lowly expressed, *CEBPB* and *CEBPG* are relatively highly expressed. Dotted line marks the median expression of all genes in the four datasets (Y = 6.19). (**B**,**C**) mRNA expression of (**B**) *CEBPB* and (**C**) *CEBPG* in microdissected tumor and stromal cells in the Renz—(black) [24] and Pilarsky—(blue) [25] dataset. Shown is the mean ± SEM. Levels of significance: ns = not significant, *** *p* < 0.001 and **** *p* < 0.0001. Abbreviations: T = tumor samples; S = stromal samples; *n* = normal tissue samples.

To assess potential redundancy and compensatory mechanisms between C/EBP-family members in PDAC, we first assessed which of the C/EBP-members are expressed in PDAC. Using publicly available mRNA-sequencing data of cell line panels, we found that *CEBPB* and *CEBPG* are highly expressed in PDAC cell lines, while *CEBPA* and *CEBPE* are rather lowly expressed (Figure 1A). Next, to elucidate the expression of the highly expressed C/EBP-family members in PDAC compared to that in healthy pancreas tissue, we queried publicly accessible, microdissected RNA-sequencing datasets for which PDAC samples, as well as stromal samples [24,25], were available (Figure 1B,C). As shown in Figure 1B, *CEBPB* expression was downregulated in the tumor samples of the Renz dataset, but it did not differ between tumor and stroma samples in the Pilarsky dataset. *CEBPG,* on the other hand, was significantly upregulated in the tumor samples of both datasets when compared to the control samples (Figure 1C). In conclusion, we found that *CEBPB* and *CEBPG* are abundantly expressed in PDAC cell lines, which, together with the finding that *CEBP*-family members are differentially regulated in tumor compared to normal tissue, suggests that *CEBPB* and *CEBPG* may have the potential to compensate for the loss of *CEBPD* in PDAC.

### 2.2. Correlation of CEBPD, CEBPB and CEBPG mRNA and Protein in PDAC Tissues

**Figure 2:**
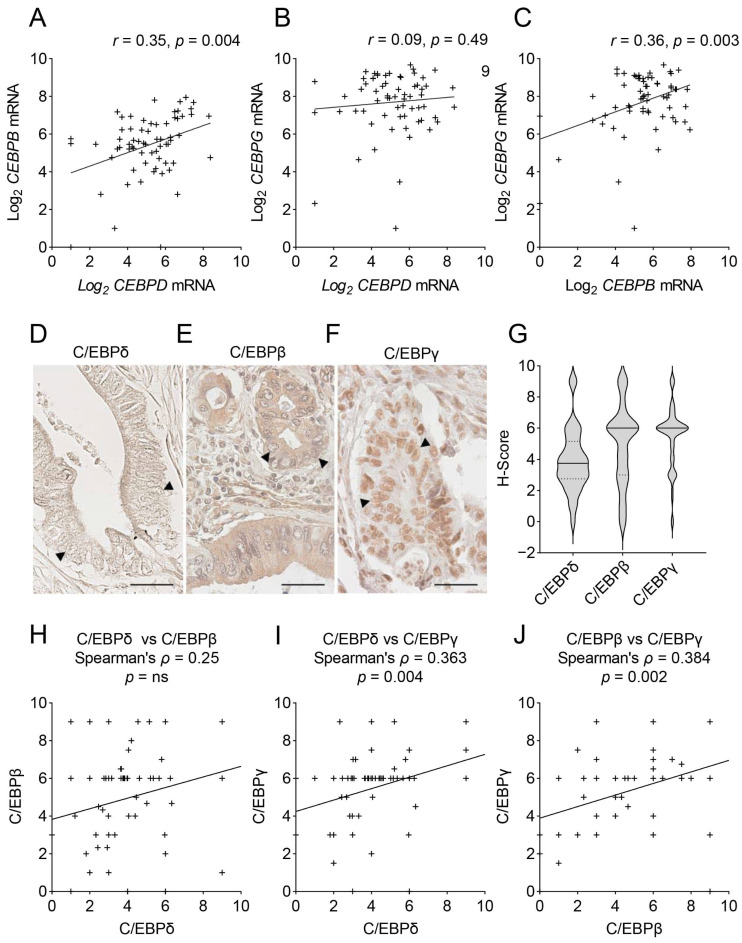
Correlations of *CEBPD*, *CEBPB* and *CEBPG* mRNA and protein expression in PDAC. (**A**–**C**) Shown are XY-plots of the correlations of (**A**) *CEBPD* vs. *CEBPB* mRNA, (**B**) *CEBPD* vs. *CEBPG* mRNA and (**C**) *CEBPB* vs. *CEBPG* mRNA in the publicly available mRNA-sequencing dataset published by Renz et al. [24]. Denoted are the Pearson correlation coefficient (*r*) and the significance (*p*) of the correlation. (**D**–**F**) Immunohistochemical staining of C/EBPδ (**D**), C/EBPβ (**E**) and C/EBPγ (**F**) in PDAC tissues. Arrow heads mark tumor cell nuclei. Scale bars = 40 µm. (**G**) Shown are the distributions of H-scores for C/EBPδ, C/EBPβ and C/EBPγ in 68 PDAC cases. Dotted lines mark the upper and lower quartiles, full line marks the median. (**H**–**J**) XY-plots show the correlation of H-scores (as shown in Figure 2G) for all three proteins against one another. Denoted are Spearman’s correlation coefficient (*ρ*) and the significance of the correlation (*p*). ’+’ signs mark individual patients in XY-plots, ‘++’ indicates two patients with similar expressions. ns = not significant.

The aim of this study was to assess potential redundancies and compensatory activities of *CEBP*-family members in PDAC. One prerequisite for redundancy and transcriptional compensation is that two genes show different expression patterns. As *CEBP*-members are often regulated similarly, we addressed this issue by investigating the correlation of each of the three *CEBP*-family members with one another in the epithelial tumor samples of publicly available, microdissected RNA-sequencing datasets. Representative plots of the Renz-dataset [24] are shown in Figure 2. From these, it becomes clear that on the mRNA level, *CEBPB* correlates with *CEBPD* and *CEBPG*. However, these correlations are not very strong and many patients fall far above or below the regression line. In addition, in further datasets presented in Table S2, *CEBPD* and *CEBPB* mRNA expression often correlate, although multiple patients expressing high levels of one and low levels of the other family member. Overall, the rather weak correlations reinforce the hypothesis that patient stratification could be improved by combining *CEBPD*, *CEBPB* and *CEBPG* expression levels. 

Although RNA-sequencing is a frequently employed method to study gene expression, mRNA expression and protein levels do not always correlate and RNA-sequencing cannot discriminate between nuclear (transcriptionally active) and cytoplasmic (transcriptionally inactive) protein levels. Thus, next, we quantified the protein expression of the three C/EBP-siblings in the tumor cell nuclei of a PDAC patient cohort of 68 patients (Figure 2D–G). While we previously described that C/EBPδ is lowly expressed in many PDAC cases [15], here we show that C/EBPβ and C/EBPγ, on the other hand, are easily detected in most tumor cell nuclei (Figure 2E,F). While C/EBPδ showed a diverse distribution of an H-score around a mean of 3.9 points (median = 3.75), the distributions of C/EBPβ and C/EBPγ were narrower and showed a marked peak at a mean H-score of 4.9 (median = 6) and 5.4 (median = 6) points, respectively (Figure 2G). Next, we tested, using the derived H-scores, whether any of the three proteins correlated with one another (Figure 2H–J). At the protein level, we found no significant correlation of C/EBPδ with C/EBPβ and only a weakly significant correlation of C/EBPγ with both C/EBPδ and C/EBPβ, with many patients expressing high levels of one proteins but low levels of the respective other protein again. These data support the possibility of mutual compensation among C/EBP-family members and might have important clinical implications.

### 2.3. C/EBPβ and C/EBPγ Partially Compensate for Low C/EBPδ to Promote Patient Survival

**Figure 3:**
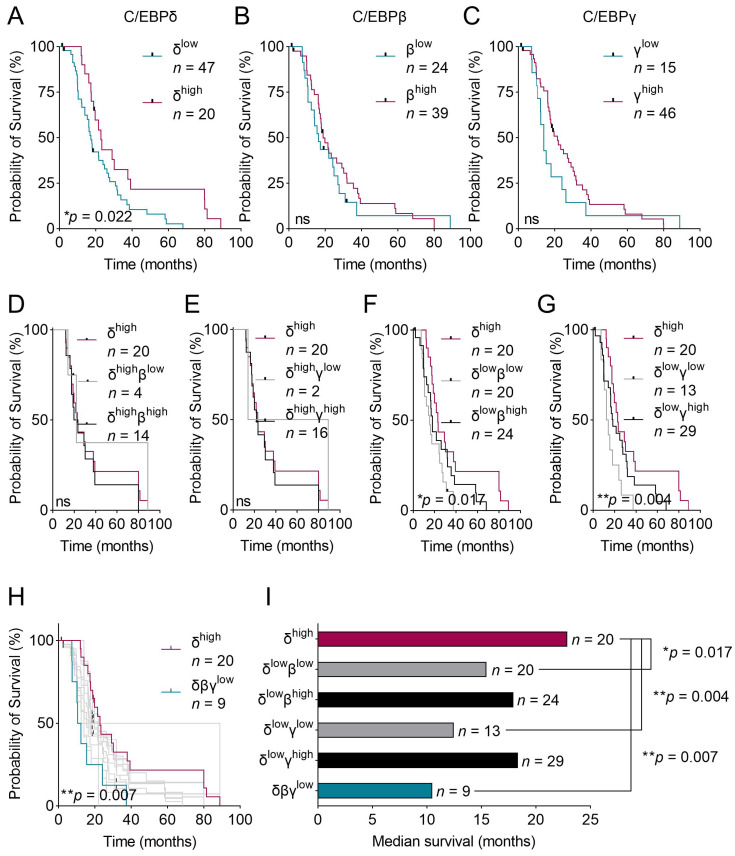
Kaplan–Meier curves of patients expressing various levels of C/EBP-proteins and those combinations. (**A**–**C**) Kaplan–Meier curves of patients with either high or low expression of (**A**) C/EBPδ, (**B**) C/EBPβ and (**C**) C/EBPγ. (**D**–**G**) Kaplan–Meier curves of patients with high C/EBPδ (red curve) as well as patients with (**D**) high C/EBPδ with low C/EBPβ (gray line) and high C/EBPδ with high C/EBPβ (black line), (**E**) high C/EBPδ with low C/EBPγ (gray line) and high C/EBPδ with high C/EBPγ (black line), (**F**) low C/EBPδ with low C/EBPβ (gray line) and low C/EBPδ with high C/EBPβ (black line), (**G**) low C/EBPδ with low C/EBPγ (gray line) and low C/EBPδ with high C/EBPγ (black line). *p*-values correspond to the survival difference measured between patients with the best survival, i.e. high C/EBPδ (red line) and patients expressing low C/EBPδ with either low C/EBPβ (**F**) or low C/EBPγ (**G**) (gray lines). (**H**) Panel H combines survival curves of all patient groups discussed in panels (**A**–**G**). *p*-value compares the survival of patients with high C/EBPδ expression (red curve) with patients having low expression of all three proteins (turquoise curve). (**I**) Plotted is the median survival of the same patient groups as shown in panels (**F**,**G**) and of patients with low expression of all three proteins. Log-rank tests were used to estimate the effect of protein expression levels on survival. ns = not significant.

We previously found that C/EBPδ protein expression correlates with prolonged patient survival in PDAC (data replicated in Figure 3A) [15]. In the present study, we expanded those findings by testing the effects of C/EBPβ and C/EBPγ protein expression on patient survival and whether C/EBPβ or C/EBPγ can compensate for lost C/EBPδ. We found that neither patients with high C/EBPβ nor with high C/EBPγ expression in primary tumor cells showed an improved median survival compared to patients with low expression of the respective protein (*p* = 0.471 and *p* = 0.307, respectively) (Figure 3B,C). Thus, neither C/EBPβ nor C/EBPγ serve as predictors of patient survival. As C/EBPδ is typically lowly expressed in PDAC, we then asked whether high C/EBPβ or high C/EBPγ expression could further improve survival in patients with still relatively high C/EBPδ as opposed to patients lacking C/EBPβ or C/EBPγ (Figure 3D,E). To this end, we plotted the survival of all patients expressing high levels of C/EBPδ (red lines) and further separated these patients by means of their C/EBPβ (Figure 3D) or C/EBPγ (Figure 3E) expression (gray lines for low expression and black lines for high expression). We did not find a significant difference in survival between the group expressing high C/EBPδ and the patients where C/EBPβ or C/EBPγ were lost, suggesting that C/EBPβ and C/EBPγ expression are irrelevant when C/EBPδ is highly expressed. Conversely, when looking at patients with low C/EBPδ expression (Figure 3F,G), and again separating these based on their C/EBPβ (Figure 3F) and C/EBPγ (Figure 3G) expression levels, we found that the groups where C/EBPδ and either C/EBPβ or C/EBPγ were lost (gray lines) showed a significantly worse survival than patients expressing high C/EBPδ (red line). Patients who had only lost C/EBPδ but retained high levels of C/EBPβ or C/EBPγ (black lines), on the other hand, showed a survival approximating that of patients expressing high levels of C/EBPδ (red line). These differences suggest that C/EBPβ or C/EBPγ can partially compensate for the loss of C/EBPδ to improve patient survival. Figure 3H shows the survival curves of all groups discussed in panels A–G. From these, it becomes clear that patients that lack all three C/EBP-members (turquoise curve) experience the worst survival rate, while patients expressing high C/EBPδ (red curve)—irrespective of C/EBPβ and C/EBPγ expression—show the best survival of all groups. Figure 3I summarizes each discussed group’s median survival. From this, it can be visually derived that the compensation of both C/EBPγ or C/EBPβ for C/EBPδ enhances patient survival when C/EBPδ is low. Successive loss of all three C/EBPs notably results in the shortest median survival of all groups analyzed.

Altogether, these findings suggest that unlike C/EBPδ, C/EBPβ and C/EBPγ do not serve as stand-alone predictors of patient survival in PDAC. Irrespectively, if C/EBPδ is lost—which occurs in many PDAC cases—C/EBPβ and C/EBPγ seem to compensate for that loss to some extent in order to promote patient survival. When both C/EBPβ and C/EBPγ are lost, however, C/EBPδ’s functions cannot be compensated anymore and patient survival decreases dramatically. This implies a partial redundancy of the three C/EBP-members and urges the analysis of both C/EBPβ and C/EBPγ, in addition to C/EBPδ, when predicting patient survival.

### 2.4. C/EBPβ Refines C/EBPδ-Based Prediction of Lymph Node-Involvement

**Figure 4:**
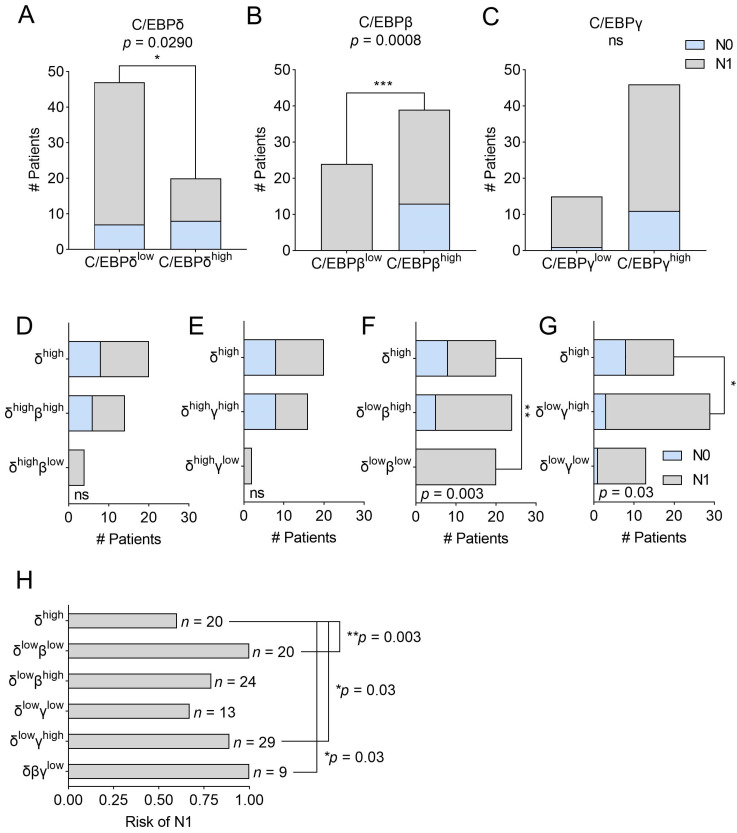
Bar graphs representing contingency tables of C/EBP-expression and lymph node involvement. Panels (**A**–**C**) Plotted are the number of patients experiencing no lymph node involvement (light blue) or lymph node involvement (gray) dichotomized by (**A**) C/EBPδ, (**B**) C/EBPβ or (**C**) C/EBPγ protein expression. Panels (**D**–**G**) show the same groups as Figure 3D–G, i.e., bar graphs of contingency tables of patients with high C/EBPδ (top bar) as well as patients with (**D**) high C/EBPδ with high C/EBPβ and high C/EBPδ with low C/EBPβ, (**E**) high C/EBPδ with high C/EBPγ and high C/EBPδ with low C/EBPγ, (**F**) low C/EBPδ with high C/EBPβ and low C/EBPδ with low C/EBPβ, (**G**) low C/EBPδ with high C/EBPγ and low C/EBPδ with low C/EBPγ. Stainings for C/EBPβ or C/EBPγ were not available for all patients in the C/EBPδ^high^ group, resulting in slightly different patient numbers among the groups. (**H**) Plotted is the risk of experiencing lymph node involvement per patient group and the number of patients per group (*n*). *p*-values are derived from Fisher’s exact tests. ns = not significant, * *p* < 0.05, ** *p* < 0.01, and *** *p* < 0.001.

Along with identifying an association with patient survival, we previously showed that PDAC patients with high C/EBPδ expression in primary tumor cells have a significantly decreased likelihood of lymph node involvement compared to patients with low C/EBPδ expression [15]. Here, we tested whether dichotomization of patients by expression of either C/EBP-family member can predict whether a patient experiences lymph node involvement and, again, whether either C/EBPβ or C/EBPγ can compensate for low C/EBPδ expression in order to prevent lymph node involvement. We confirmed that low primary tumor cell C/EBPδ increases the likelihood of lymph node involvement (*p* = 0.029) (Figure 4A) and further found that high C/EBPβ, but not high C/EBPγ alone, is associated with a decreased likelihood of lymph node involvement (*p* = 0.0008 and *p* = 0.261, respectively) (Figure 4B,C). Notably, low levels of C/EBPβ protein in primary tumor cells showed an even stronger association with lymph node involvement than low expression of C/EBPδ. 

Next, we assessed whether C/EBPβ or C/EBPγ could also compensate for C/EBPδ in lymph node involvement. To this end, the same groups as shown in Figure 3D–G were analyzed. In Figure 4D,E, we plotted all patients expressing high C/EBPδ irrespective of their C/EBPβ or C/EBPγ expression (top bar) and show the number of patients experiencing lymph node involvement (gray fraction) or no lymph node involvement (light blue fraction). We then split patients according to high or low C/EBPβ (Figure 4D) or C/EBPγ (Figure 4E) expression and found that the level of either C/EBPβ or C/EBPγ expression had no significant influence on lymph node involvement when C/EBPδ is highly expressed. Notably, stainings for C/EBPβ or C/EBPγ were not available for all patients in the C/EBPδ^high^ group, resulting in slightly different patient numbers among the groups. Next, in Figure 4F,G, we plotted the patients expressing high C/EBPδ (top bar) next to patients expressing low C/EBPδ, together with either high or low C/EBPβ (Figure 4F) or C/EBPγ (Figure 4G), to assess at the effect of C/EBPβ and C/EBPγ when C/EBPδ is nearly absent. Here, we found that patients expressing low levels of C/EBPδ and C/EBPβ (Figure 4F, lowest bar) have a significantly higher likelihood of developing lymph node involvement compared to patients expressing high C/EBPδ (*p* = 0.003) (Figure 4F, top bar). This difference is diminished when C/EBPβ is still expressed (Figure 4F, middle bar), implying that the expression of C/EBPβ can partially compensate for the lack of C/EBPδ to prevent lymph node involvement. Interestingly, when employing the same analytic approach vice versa, we found that C/EBPδ can also compensate for low expression of C/EBPβ (*p* = 0.0025 for C/EBPβ^high^ vs. C/EBPβ^low^ + C/EBPδ^low^ and *p* = 0.29 for C/EBPβ^high^ vs. C/EBPβ^low^ + C/EBPδ^high^). In contrast, when testing the effect of C/EBPγ expression in patients with low C/EBPδ (Figure 4G), we found that high C/EBPγ promotes lymph node involvement (*p* = 0.03) in the near absence of C/EBPδ. Figure 4H shows the risk of the respective patient groups as regards having lymph node involvement and clearly visualizes that a loss of C/EBPβ in addition to low C/EBPδ results in a significantly increased risk of having tumor cell-positive lymph nodes (risk = 1 vs. 0.6 for C/EBPδ^high^-patients). This risk decreases when C/EBPβ is highly expressed (risk = 0.79), implying that C/EBPβ might indeed compensate for the loss of C/EBPδ in this context.

Thus, it appears that along with C/EBPδ, C/EBPβ is a major determinant of a patient’s likelihood to experience lymph node involvement and gives even stronger predictions about this clinical feature. While C/EBPδ and C/EBPβ can compensate for each other, C/EBPγ antagonizes C/EBPδ in this respect. Thus, analyzing the expression of all three C/EBP-members in primary tumor tissues might be clinically relevant to determine the likelihood of lymph node involvement and treatment options.

## 3. Discussion

PDAC is a dismal disease with a poor clinical prognosis and treatment options are unsatisfactory, especially for patients with advanced disease. We previously found a promising correlation of C/EBPδ protein expression in primary PDAC cell nuclei and patient survival as well as lymph node involvement. The aim of this study was to evaluate whether three major C/EBP-members act redundantly in PDAC and whether the quantification of C/EBPβ and/or C/EBPγ, in addition to C/EBPδ protein expression, in primary tumor tissue of PDAC patients might refine the prediction of patient survival or the likelihood of lymph node involvement.

C/EBPγ is especially well-studied in acute myeloid leukemia (AML), where it promotes cancer progression through the upregulation of EIF4BP1 [30]. Similarly, in esophageal squamous cell carcinoma (ESCC), C/EBPγ promotes proliferation and migration through the upregulation of the PI3K-Akt signaling pathway and serves as a prognostic marker for poor survival [31]. Thus, C/EBPγ is generally perceived as a tumor promoter and the observation that *CEBPG* mRNA is upregulated in tumor as opposed to normal PDAC tissue encourages this impression. Interestingly, when dichotomizing a cohort of 68 PDAC patients according to primary tumor cell C/EBPγ expression, patients with high C/EBPγ expression did not experience a decreased survival but rather a prolongation of 7.52 months compared to patients with low C/EBPγ expression, although this difference lacks significance. This leads to the impression that C/EBPγ expression is irrelevant for survival in most PDAC patients.

C/EBPβ has also been studied in different cancers and has, for instance, been associated with a decrease in cell proliferation and partial reduction in BRAF-inhibitor resistance in malignant melanoma [32]. In PDAC, C/EBPβ cooperates with Menin (*MEN1*) to induce cyclin-dependent kinase inhibitor 2A (*CDKN2A)* transcription and to antagonize epithelial to mesenchymal transition [33]. In breast cancer, on the other hand, C/EBPβ was associated with an increase in Claudin-4 (*CLDN4*) to promote cancer cell migration and invasion [34]. Thus, just as C/EBPδ, C/EBPβ has the status of a context-dependent player in carcinogenesis. In PDAC, we found that dichotomization of patients according to primary tumor cell C/EBPβ expression did not show a survival advantage of patients with either high or low expression of C/EBPβ, implying that C/EBPβ also does not actively regulate survival in most patients. Thus, C/EBPδ remains the strongest predictor for prolonged patient survival.

Interestingly, however, when C/EBPδ is lost, the additional loss of either C/EBPβ or C/EBPγ dramatically reduced median survival as compared to patients where C/EBPβ or C/EBPγ were still expressed. Both C/EBPβ and C/EBPγ could, thus, partially compensate for the lack of C/EBPδ and prolong patient survival. This notion of redundancy among C/EBP-family members in PDAC is reinforced by the fact that patients lacking expression of all three proteins experience the worst median overall survival of all possible combination. As C/EBPδ is lost in many PDAC patients, it would be of great clinical value to not only assess C/EBPδ expression in primary tumor tissues, but also that of C/EBPγ, and especially C/EBPβ, to predict patient survival more accurately.

Considering the role and redundancy of C/EBP-family members in lymph node involvement in PDAC, we found that C/EBPβ acts as a major predictor of a patient’s likelihood of having tumor cell-positive lymph nodes. Herein, the predictive power of C/EBPβ is even stronger than that of C/EBPδ alone. Interestingly, the two proteins might partially compensate for the lack of each other to decrease the risk of lymph node involvement. This constitutes a bi-directional compensatory mechanism as opposed to the presumably one-directional compensation of C/EBPβ for C/EBPδ in the context of patient survival. Generally, these data hint upon a tumor suppressor role of C/EBPβ in PDAC, which is further in accordance with the findings of Cheng et al., who assigned an EMT-suppressive role to C/EBPβ in PDAC [31]. Therefore, immunohistological quantification of C/EBPβ, in addition to C/EBPδ, might be of great clinical value to determine treatment options.

The finding that high expression of C/EBPγ increased the risk of lymph node involvement in the near absence of C/EBPδ implies that C/EBPγ antagonizes C/EBPδ in this context. While lymph node involvement is already enhanced under low C/EBPδ, this propensity is even reinforced by the high expression of C/EBPγ. However, to assign a tumor-promoting role to C/EBPγ in PDAC, this observation must be tested in a larger patient cohort.

It has been described before that C/EBP-family members act redundantly and can compensate for each other due to their conserved structure and DNA-binding domain. In lymphoblast cells for instance, C/EBPα, C/EBPβ and C/EBPδ individually induced LPS-induced transcription of IL-6 (*IL6*) and MCP-1 (*CCL2*), albeit at varying efficiencies [19]. In mice, knock-out of either C/EBPδ or C/EBPβ partially reduced lipid droplet formation in differentiating adipocytes, while in double knockout mice, differentiation of pre-adipocytes was blocked and no lipid droplets were formed in brown fat tissues [20]. Further, it has been suggested that C/EBPδ and C/EBPβ may compensate for the secretion of pro-inflammatory cytokines in macrophages [21,35]. Next to this, C/EBP-members are often similarly regulated, as has been shown, for instance, during the acute phase response for IL-1b-inducible C/EBPβ and C/EBPδ [22] and in AML for 1α,25-dihydroxyvitamin-induced C/EBPβ and C/EBPδ, as well as all-trans-retinoic acid-induced C/EBPβ and C/EBPε [23], further implying a synergistic behavior and potential redundancy of the C/EBP-family members. In our previous work, we studied the mechanism through which C/EBPδ might reduce the formation of lymph node metastases and found that it regulates gene signatures involved with cytoskeletal dynamics and cell motility. Further, C/EBPδ re-activated E-cadherin (CDH1), which presumably led to a partial reversion of an epithelial-to-mesenchymal (EMT) phenotype. Given the described redundancies between C/EBPδ and C/EBPβ and the strong correlation of C/EBPβ expression levels with lymph node involvement, it might thus be possible that C/EBPβ regulates mechanisms similar to those of C/EBPδ when compensating for its low expression. C/EBPγ, on the other hand, has mostly been shown to act as a negative regulator of transcription as it lacks the transactivation domain, which is in most—yet not all—cases required to initiate transcription upon dimerization with other C/EBP-members [18,36]. High levels of C/EBPγ do not affect the risk for lymph node involvement when C/EBPδ is abundant. However, when C/EBPδ is decreased, the surplus of C/EBPγ monomers presumably captures the remaining C/EBPδ monomers through dimerization and prevents the activation of important C/EBPδ-target genes regulating migration and invasion, thereby potentially contributing to lymph node involvement. Although the underlying mechanisms of C/EBP-redundancy in PDAC have not been unraveled yet, compensatory mechanisms of C/EBP-family members in PDAC seem likely. A deeper understanding of these redundancies would not only benefit the prediction of PDAC patient survival and lymph node involvement but could refine the way C/EBP-proteins are analyzed generally, namely as a family of transcription factors rather than stand-alone proteins.

Although it provides interesting insights concerning the redundancy of C/EBP-proteins in PDAC, this study is not free of limitations. The marked peaks in the distribution of C/EBPβ and C/EBPγ protein scores might be due to an actual narrow distribution but could also be attributed to the rather small number of patients included in this cohort. The limited number of patients also leads to very small groups, such as those expressing high levels of C/EBPδ with either low C/EBPβ (*n* = 4) or C/EBPγ (*n* = 2), aggravating firm conclusions about the actual survival of such groups and their risk of lymph node involvement. Further, we found that C/EBPβ is a major predictor of lymph node involvement but does not correlate with patient survival by itself. This is surprising as lymph node status has been shown to act as a predictor of overall and disease-free survival in PDAC [37]. While lymph node involvement has a binary outcome, survival depends on multiple factors, one of which is lymph node involvement. The strong association of C/EBPβ with lymph node involvement hints upon a role of C/EBPβ in predicting patient survival in large cohorts. Lastly, three more members of the C/EBP-family have been described in literature and are under investigation in the context of health and disease. Those are C/EBPα, C/EBPε and C/EBPζ. Although these proteins are either extremely lowly expressed in PDAC (C/EBPα and C/EBPε) or do not function transcriptionally activating (C/EBPζ), the presence of and interaction with these family members might have additional effects on the actions of C/EBPδ, C/EBPβ and C/EBPγ. For a more holistic analysis, those might be added in future studies.

## 4. Materials and Methods

### 4.1. Tissue Microarray (TMA)

As described before [15], 68 formalin-fixed, paraffin-embedded punches from PDAC resection specimens collected between the years of 1983 and 2015 were selected from the archives of the Pathology Department of the Amsterdam University Medical Center and combined in a tissue microarray. Patients with a known previous malignancy in another organ were excluded from the analysis. The present study included 49 men and 19 women with ages ranging from 47 to 83 with a mean (±SD) of 64 (±9.15) and a median of 63 years (Table S1). For 44 patients, 3 cores were available, and for 24 patients only 1 core was available.

### 4.2. Immunohistochemistry

First, 4 µm thick TMA cores were deparaffinized and endogenous peroxidase activity was blocked using 0.3% H_2_O_2_ in methanol for 15 minutes. Next, slides were blocked using Ultra V Block (#TA-125-UB, Thermo Fisher Scientific, Waltham, MA, USA) for 1 hour at room temperature. Slides were then incubated with primary antibodies against C/EBPβ or C/EBPγ in PBS at 4 °C overnight (C/EBPβ 1:500 #GTX100675, GeneTex, Irvine, CA, USA; C/EBPγ 1:500 #abx103894, Abbexa Ltd., Cambridge, UK). The C/EBPβ antibody binds to the transcriptionally active C/EBPβ isoforms LAP and LAP* but not to the inactive isoform LIP. Only one isoform C/EBPδ and C/EBPγ is known to date. The next day, slides were incubated with secondary HRP-linked goat-anti-rabbit antibody (#DPVO55HRP, ImmunoLogic, Duiven, NL, USA) for 30 minutes at room temperature and stained using 3,3′Diaminobenzidine (Bright DAB #BS04-999, ImmunoLogic) with hematoxylin (1:10 in demineralized H_2_O) counterstain. To assess C/EBPδ expression, the TMA and scoring published previously were employed [15].

### 4.3. Quantification of Protein Expression Levels

C/EBPδ expression was scored for 67 patients, C/EBPβ staining for 63 and C/EBPγ staining for 61 patients. Due to technical issues in the preparation process, tissues from a few patients were missing from some TMA slides, leading to different numbers of patients for the different stainings. Protein expression, denoted as histology (H)-score, was assessed using a blinded semi-quantitative method in which 1–3 cores per patient were scored with respect to the DAB signal intensity (0 = negative, 1 = weak, 2 = intermediate, 3 = strong staining) and percentage of stained tumor cell nuclei (0 = 0–25%, 1 = 26–50%, 2 = 51–75% and 3 = 76–100% stained tumor cell nuclei). The intensity and percentage scores were multiplied to reach the final H-score per patient, ranging from 0 to 9. To dichotomize patients according to protein expression, patients were split into high-expression and low-expression groups for each respective protein at an H-score of 4.5. This cut-off was chosen based on the range of expression (0–9 points) and to be consistent with our previous work on C/EBPδ [15].

### 4.4. Mining of Publicly Available RNA-Sequencing Datasets

RNA-sequencing datasets were derived from Gene Expression Omnibus [38] and ArrayExpress [39] using the Genomics Analysis and Visualization Platform R2 [40]. Cell line *CEBP*-expression levels were derived from the cancer cell line encyclopedia (CCLE) (accession no. GSE36133) [26] and from datasets published by Garnett et al. (accession no. E-MTAB-783) [27], Wappett et al. (accession no. GSE57083) [28] and Maupin et al. (accession no. GSE21654) [29], whereby only cell lines with an annotated primary site as ‘pancreas’ were included. Gene expression levels of *CEBPD*, *CEBPB* and *CEBPG* in pancreatic tumor and normal tissue were derived from publicly available microdissected RNA-sequencing datasets published by Renz et al. (accession no. GSE93326) [24] and Pilarsky et al. (accession no. E-MEXP-1121) [25]. *CEBPD*, *CEBPB* and *CEBPG* mRNA expression levels in tumor samples were derived from Janky et al. (accession no. GSE62165) [41], Stratford et al. (accession no. GSE21501) [42], Renz et al. (accession no. GSE93326) [24], Perez-Mancera et al. (accession no. GSE36924) [43], Zhao et al. (accession no. GSE184585) [44], Bailey et al. (accession no. GSE36924) [45], and Guo et al. (accession no. GSE172356) [46]; in the TCGA pancreatic cancer dataset, wherefrom non-tumor samples were excluded, only samples with a tumor cell content of more than 30% were included.

### 4.5. Statistical Analyses

Gene expression levels of CEBP-family members in PDAC cell lines were derived from the Genomics Analysis and Visualization Platform R2 [40] and graphs were prepared using GraphPad Prism (version 9.1.0, GraphPad Software Inc., San Diego, CA, USA). Gene expression levels of *CEBPD*, *CEBPB* and *CEBPG* in pancreatic tumor and normal tissue were compared using one-way ANOVA. Where data were normally distributed, Pearson correlation was used to calculate correlation coefficients (R) and the significance of correlations of *CEBPD*, *CEBPB* and *CEBPG* mRNA in tumor cells specifically; otherwise, Spearman’s rank correlation was used. Both ANOVAs and Pearson correlations were performed in the Genomics Analysis and Visualization Platform R2 [40], while Spearman rank correlation was performed using GraphPad Prism. Correlations of protein expression levels were assessed using Spearman’s rank correlation and performed using the statistical software suite SPSS (IBM SPSS Statistics for Windows, Version 26.0. 2019, Armonk, NY, USA) [47]. Moreover, Log-rank tests and Mann–Whitney U tests comparing protein expression between patients with and without lymph node involvement were performed using SPSS [47]. The effects of C/EBP-proteins’ expression levels on lymph node involvement were analyzed using contingency tables and Fisher’s exact test in SPSS [47]. Fisher’s exact tests were performed and all graphs were compiled using GraphPad Prism.

## 5. Conclusions

In this study, using a cohort of 68 PDAC patients, we found that C/EBPβ and C/EBPγ expression in primary tumor cells can partially compensate for the frequent loss of tumor cell C/EBPδ in order to improve patient survival. We further found that low C/EBPβ protein expression acts as a novel predictor of lymph node involvement in PDAC. Both C/EBPβ and C/EBPδ could compensate for the lack of each other to reduce the risk of lymph node involvement. High levels of C/EBPγ, on the other hand, increased the risk of lymph node involvement in patients where C/EBPδ was decreased. Thus, the redundancies among C/EBP-family members might have a profound influence on clinical prognoses and the expression of both C/EPBβ and C/EBPγ should be taken into account when dichotomizing patients according to C/EBPδ expression.

## Data Availability

Publicly available datasets were analyzed in this study. This data can be found here: https://www.ncbi.nlm.nih.gov/geo/, accession numbers GSE9336 [24], GSE71729 [26], GSE36133 [27], GSE57083, GSE21654 [30], GSE62165 [42], GSE21501 [43], GSE36924 [44], GSE184585 [45], GSE36924 [45], GSE172356 [46] and https://www.ebi.ac.uk/biostudies/arrayexpress, accession numbers E-MEXP-1121 [25] and E-MTAB-783 [28] (last accessed on 1 November 2022).

## Supplementary Materials

The following supporting information can be downloaded at: https://www.mdpi.com/article/10.3390/ijms24021537/s1. References [34,35,36,37,38,39] are cited in Supplementary Materials.
