# Supplementary material for: C/EBP-Family Redundancy Determines Patient Survival and Lymph Node Involvement in PDAC
Source: Int J Mol Sci. 2023 Jan 12;24(2):1537. doi: 10.3390/ijms24021537 (PMC9867044; doi:10.3390/ijms24021537)
Supplement: Supplementary file 1 [file ijms-24-01537-s001.zip › ijms-2057424-supplementary.pdf]

Supplementary Data

Table S1: Characteristics of patients included in the cohort.

Characteristic	all patients (N=68)		C/EBPδ^{low} (N=47)		C/EBPδ^{high} (N=20)	
	N	%	N	%	N	%
Median age	64.01		66		61	
(range) (years)	(47-83)		(47-82)		(48-83)	
Sex (M/F)	(49/19)	(72.1/27.9)	(34/13)	(72.3/27.6)	(15/5)	(75/25)
Surgery						
PPPD	60	88.2	40	85.1	19	95
Whipple-Kausch	8	11.8	7	14.9	1	5
Radicality						
R0 (≤1mm)	32	47.1	21	44.7	11	55
R1 (≥1mm)	27	39.7	20	42.6	7	35
Dubious	8	11.8	6	12.8	2	10
N/A	1	1.5	0	0.0	0	0
Diameter post-op (cm)						
1-2	2	2.9	1	2.1	0	0
2-4	34	50.0	25	53.2	9	45
4-6	17	25.0	10	21.3	7	35
N/A	15	22.1	11	23.4	4	20
N-Stage						
N0	15	22.1	7	14.9	8	40
N1	53	77.9	40	85.1	12	60
Grading						
Well differentiated	2	2.9	1	2.1	1	5
Moderately differentiated	18	26.5	12	25.5	6	30
Poorly differentiated	18	26.5	13	27.7	5	25
Undifferentiated	3	4.4	2	4.3	1	5
N/A	27	39.7	19	40.4	7	35
Survival						
Median	18.89		17.35		22.9	
(range) (months)	(1.58-88.9)		(1.58-68.11)		(12.02-88.9)	

Table S1 (continued)

Characteristic	C/EBPβ^{low} (N=24)		C/EBPβ^{high} (N=39)	
	N	%	N	%
Median age	68		63	
(range) (years)	(48-82)		(47-83)	
Sex (M/F)	(18/6)	(75/25)	(29/10)	(74.4/25.6)
Surgery				
PPPD	23	95.8	33	84.6
Whipple-Kausch	1	4.2	6	15.4
Radicality				
R0 (≤1mm)	11	45.8	19	48.7
R1 (≥1mm)	10	41.7	15	38.5
Dubious	3	12.5	4	10.3
N/A	0	0.0	1	2.6
Diameter post-op (cm)				
1-2	1	4.2	1	2.6
2-4	13	54.2	19	48.7
4-6	6	25.0	11	28.2
N/A	4	16.7	8	20.5
N-Stage				
N0	0	0.0	13	33.3
N1	24	100.0	26	66.7
Grading				
Well differentiated	0	0.0	2	5.1
Moderately differentiated	11	45.8	6	15.4
Poorly differentiated	8	33.3	10	25.6
Undifferentiated	0	0.0	3	7.7
N/A	5	20.8	18	46.2
Survival				
Median	16.33		18.89	
**(range) (months)**	(1.58-88.9)		(2.1-80)	

Table S1 (continued)

Characteristic	C/EBPγ^low (N=15)		C/EBPγ^high (N=46)	
	N	%	N	%
Median age	67		63	
(range) (years)	(53-78)		(47-83)	
Sex (M/F)	(13/2)	(86.7/13.3)	(32/14)	(69.6/30.4)
Surgery				
PPPD	15	100.0	39	84.8
Whipple-Kausch	0	0.0	7	15.2
Radicality				
R0 (≤1mm)	10	66.7	19	41.3
R1 (≥1mm)	4	26.7	20	43.5
Dubious	1	6.7	6	13.0
N/A	0	0.0	1	2.2
Diameter post-op (cm)				
1-2	1	6.7	1	2.2
2-4	7	46.7	25	54.3
4-6	6	40.0	11	23.9
N/A	1	6.7	9	19.6
N-Stage				
N0	1	6.7	11	23.9
N1	14	93.3	35	76.1
Grading				
Well differentiated	1	6.7	1	2.2
Moderately differentiated	9	60.0	8	17.4
Poorly differentiated	3	20.0	15	32.6
Undifferentiated	0	0.0	3	6.5
N/A	2	13.3	19	41.3
Survival				
Median	14		21.52	
(range) (months)	(1.58-88.9)		(2.1-80)	

Table S2: Correlations of *CEBPD*, *CEBPB* and *CEBPG* mRNA in publicly available RNA-sequencing datasets. Denoted are the correlation coefficient (r) and the significance of the correlation. Significant correlations are denoted in bold letters.

Data Set	***CEBPD* vs *CEBPB***	***CEBPD* vs *CEBPG***	***CEBPB* vs *CEBPG***
Janky et al. [34] (N=118)	***r*=0.413, *p*=0.000**	***r*=-0.235, *p*=0.007**	***r*=-0.261, *p*=0.003**
TCGA (N=21)	***r*=0.801, *p*=0.000**	***r*=-0.557, *p*=0.006**	*r*=-0.421, *p*=0.058
Stratford et al. [35] (N=131)	***r*=0.178, *p*=0.042**	***r*=-0.187, *p*=0.033**	*r*=0.083, *p*=0.343
Perez-Mancera et al. [36] (N=91)	***r*=0.497, *p*=0.000**	*r*=-0.227, *p*=0.030	*r*=-0.195, *p*=0.064
Zhao et al. [37] (N=82)	***r*=0.582, *p*=0.000**	***r*=0.411, *p*=0.000**	***r*=0.419, *p*=0.000**
Bailey et al. [38] (N=70)	***r*=0.366, *p*=0.002**	*r*=-0.119, *p*=0.327	*r*=-0.020, *p*=0.820
Guo et al. [39] (N=62)	***r*=0.626, *p*=0.000**	*r*=-0.215, *p*=0.093	*r*=0.0850, *p*=0.497
